# A Review of Processing Techniques and Rheological Properties of Yogurts

**DOI:** 10.1111/jtxs.70006

**Published:** 2025-02-05

**Authors:** Thong Le Ba, Mai Sao Dam, Lien Le Phuong Nguyen, László Baranyai, Tímea Kaszab

**Affiliations:** ^1^ Institute of Food Science and Technology, Hungarian University of Agriculture and Life Sciences Budapest Hungary; ^2^ Industrial University of Ho Chi Minh City Ho Chi Minh Vietnam

**Keywords:** dairy products, microstructure, Newtonian liquid, thixotropic gel

## Abstract

The rheology of yogurt, a critical factor influencing its texture, stability, and sensory appeal from production to consumption is explored in this review. Yogurt undergoes a transformation from a Newtonian liquid to a non‐Newtonian thixotropic gel during fermentation, a process shaped by factors such as milk source, starter culture, and fat content. Recent advances in non‐thermal treatments, such as ultrasound, microfiltration, high‐pressure processing (HPP), and ultraviolet C (UV‐C) light treatment, promise to enhance nutritional and sensory qualities of yogurt. Additionally, incorporating herbs and fruits into yogurt not only diversifies consumer options but also affects its texture, viscosity, and overall mouthfeel. The review examines how different yogurt styles—set, stirred, Greek, and drinkable—develop unique textures through varied production processes. Advances in rheological techniques and microstructural analysis have deepened our understanding of the interactions between proteins and fat globules, providing insights into the complex gel network of yogurt. Despite significant progress, further research is needed in areas such as non‐destructive rheological testing during production, the impact of extended storage, and the development of plant‐based yogurt alternatives. The insights are valuable for the dairy industry to support innovation and meet growing consumer demands.

## Graphical abstract

This review explores the rheology of yogurt, highlighting its transformation from Newtonian fluid to non‐Newtonian gel. Key factors such as milk source, starter culture and fat content are examined along with non‐thermal treatments (ultrasound, HPP). Advanced techniques will provide insight into the texture, stability and sensory properties of yogurt.

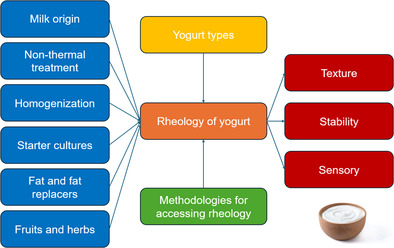

## 1 Introduction

Yogurt, traditionally a fermented milk product, can be derived from animal sources, such as cow, sheep, and camel milk, as well as plant‐based alternatives like almond, coconut, and oat milk. This diversity is in response to growing consumer demand for lactose‐free and vegan options (Lucey, and Singh 1997; Peng et al. 2009; Wang et al. 2025. Its ability to incorporate probiotics enhances its nutritional and physiological value (Loveday, Sarkar, and Singh 2013; Dantas et al. 2016). Beyond the health benefits associated with dairy products, the texture and taste of yogurt play essential roles in shaping consumer preferences. In the dairy industry, microorganisms such as *Lactobacillus delbrueckii subsp. bulgaricus* and *Streptococcus thermophilus* are primary starter cultures in yogurt production. Secondary or optional cultures include *Lactobacillus casei* , *Lactobacillus lactis* , *Lactobacillus jugurtiticus*, *Bifidobacterium longum* , *Bifidobacterium bifidum* , *Bifidobacterium infantis* , and *Lactobacillus acidophilus* (Vedamuthu 2013; Dönmez, Mogol, and Gökmen 2017). These strains produce various compounds such as exopolysaccharides, lactic acid, and acetaldehyde, which contribute to the consistency, flavor, texture, and color of yogurt (Charchoghlyan et al. 2017; Chen et al. 2017).

Yogurt is a nutrient‐dense food that supports digestive health, including for individuals with lactose intolerance, and contributes to overall cardiovascular wellness (Chandan, Gandhi, and Shah 2017; Hadjimbei, Botsaris, and Chrysostomou 2022). The market offers a diverse range of yogurt options, starting from the general set, stirred, drinking, until the specific walnut, Greek, high‐protein types, enhancing its popularity (Yildiz 2009; Chandan and Nauth 2012; Hill et al. 2017). There are three main categories of yogurt: drinking, set, and stirred (Karam et al. 2013). Set and stirred yogurts differ in the filling step, which occurs before or after incubation, while stirred yogurt can be diluted to make drinking yogurt (Shah 2003; Arab et al. 2019).

The starter culture plays a crucial role in milk fermentation by gradually lowering the pH through lactic acid production. As the pH decreases, casein proteins aggregate, forming a fragile gel network (De Brabandere and De Baerdemaeker 1999; McCann, Fabre, and Day 2011; Cui, Lu, Tan, Wang, and Li, 2014). The decrease in steric stabilization and electrostatic repulsions, along with increased interactions between caseins, results in a network of interconnected strands that trap water, lactose, whey proteins, and fat globules, creating a gel (Meletharayil, Patel, and Huppertz 2015; Das, Choudhary, and Thompson‐Witrick 2019; Bai et al. 2020). Set yogurt is characterized by this stable gel network, whereas stirred yogurt is produced by mechanically disrupting the gel network of set yogurt, resulting in a product with a different texture and consistency. Subsequent operations, such as mixing, pumping, and packing, affect the structure, leading to a decrease in apparent viscosity (η) (Espírito‐Santo et al. 2013). The flow characteristics of the final protein network determine whether yogurts are set, drinking, or stirred products (Yoon and McCarthy 2002).

The rheological, microstructural, and textural properties of yogurt are influenced by a variety of factors, including milk origin, non‐thermal treatments, starter culture, milk compounds like fat, fat replacers, and other added ingredients such as whey protein and fruits (Jaros et al. 2002; Amatayakul, Sherkat, and Shah 2006; Tamime and Robinson 2007a; Riener et al. 2010; Delikanli and Ozcan 2014; Puvanenthiran et al. 2014). Milk origin determines the composition of proteins and fats, affecting the density and arrangement of casein micelles and fat globules, thereby influencing gel structure and viscosity of yogurt. Non‐thermal treatments during milk pasteurization, such as HPP or microfluidization, can induce structural changes in proteins and fats, which then affect the gel network formation and overall texture of yogurt. Moreover, the choice of starter culture impacts the fermentation process, influencing acidification rates, enzyme activities, and EPS production, which in turn affect the gel structure and texture (Jaros et al. 2002; Amatayakul, Sherkat, and Shah 2006). In addition, fat content directly affects the mouthfeel and creaminess of yogurt, with higher fat content generally resulting in a richer texture (Lee and Lucey 2006; Cayot et al. 2008). Fat replacers, such as modified starch or inulin, can alter the rheological properties by mimicking the functionality of fat globules or affecting water binding capacity (Haque, Richardson, and Morris 2001; Damin et al. 2009). On the other hand, homogenization modifies the size and distribution of fat globules, leading to changes in viscosity, texture, and stability, while the addition of fruits or herbs introduces additional solids and sugars, impacting texture, flavor, and viscosity (Sameen et al. 2010).

Methodologies for measuring the rheological properties of yogurt encompass a range of techniques, from simple viscosity measurements to more sophisticated techniques like oscillatory rheometry and texture profile analysis (Karagül‐Yüceer and Drake 2013; Mudgil, Barak, and Khatkar 2017; Upadhyay and Chen 2020). These methods help quantify parameters such as viscosity, yield stress ($\tau_{\text{yield}}$), thixotropy, and viscoelasticity. Each of these factors contributes to the complex matrix of yogurt, influencing its sensory attributes and consumer acceptance. This article focuses on understanding the rheology of yogurt and explores the importance of these insights for both industry and consumers.

## 2 Rheology of Yogurt

The term “rheological properties” broadly captures the sensory and flow‐related characteristics of yogurt, including its microstructure, mouthfeel, texture, and body. While “texture” and “body” are often used interchangeably, they have distinct meanings. The overall physical structure of yogurt, encompassing its bulk, is typically referred to as its “body”. This attribute can be characterized using terms such as weak, lumpy, gel‐like, or firm (Tribby 2008). It is important to note that “hardness,” frequently used in yogurt texture analysis, is not entirely suitable since hardness applies to solid foods, while yogurt is semi‐solid. Therefore, “firmness” is more appropriate in this context.

In yogurt, “texture” refers to its microstructure and the arrangement of its tiny parts. According to Szczesniak (2002), texture is defined more broadly as the functional experience and sensory perception of the structure, mechanics, and surface characteristics of food, perceived through kinesthetics, touch, hearing, and vision. Meanwhile, yogurt rheology is the scientific study of how yogurt gel flows and deforms under both tangential and normal stress. Exploring the rheology of yogurt provides insight into its structural and mechanical properties, shedding light on its behavior under various conditions and pressures.

Shear stress represents the force applied to the cross‐section of yogurt, while shear rate is how fast the force is applied, and stress–strain shows how much the yogurt deforms and changes shape. Key rheological parameters (Lubbers et al. 2004) include the consistency index, η, storage modulus ($G^{\prime }$), and loss modulus ($G^{"}$). $G^{\prime }$ represents the energy stored in the material, reflecting its elastic properties while $G^{"}$ represents the energy dissipated as heat, reflecting its viscous properties.

**FIGURE 1:**
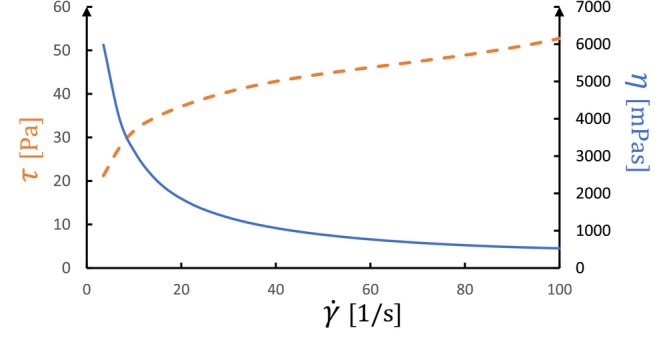
Flow curve of yogurt. *Source:* Self‐measured.

Viscosity describes how much a liquid resists flow caused by friction between its moving particles. Unlike water, a simple “Newtonian” liquid, yogurt exhibits complex behavior, being viscoelastic, shear‐thinning, and thixotropic (Lubbers et al. 2004; Damin et al. 2009; Lee and Lucey 2010). The term “viscoelastic” describes s that combine the elasticity of solids (tendency to return to their original shape) with the viscosity of liquids (tendency to flow under stress). The change in particle orientation triggered by increased shear rates is responsible for the reduction in internal friction observed in shear‐thinning materials like yogurt, meaning its viscosity decreases with increasing shear rate, as shown in Figure 1. Thixotropy is characterized by a continuous reduction in apparent viscosity when subjected to constant shear over time. Once the shear stops, the viscosity recovers, although yogurt thixotropy is partial because the structural breakdown caused by shear isn't entirely reversible (Lee and Lucey 2010).

The partitioning of energy between elastic storage and flow dissipation in viscoelastic materials such as yogurt under stress is important for understanding its behavior. Quantifying this balance through moduli ($G^{\prime },G^{"}$) is crucial for characterizing various properties of yogurt. For instance, in the transition from liquid to gel, gelation is indicated when $G^{\prime }$ exceeds 1 Pa. $\sigma_{\text{yield}}$, marked by a decrease in moduli values, signifies the onset of flow and can be identified by a declining shear stress curve (Lee and Lucey 2006). High‐loss tangent (δ) values characterize weak, fluid‐like gels, while low δ values indicate firm, rubbery gels (Hovjecki et al. 2021). Determining important rheological parameters during yogurt gelation helps understand its structural transition, contributing to the optimization of product texture and stability (Bankole et al. 2023).

## 3 Production of Yogurt

**FIGURE 2:**
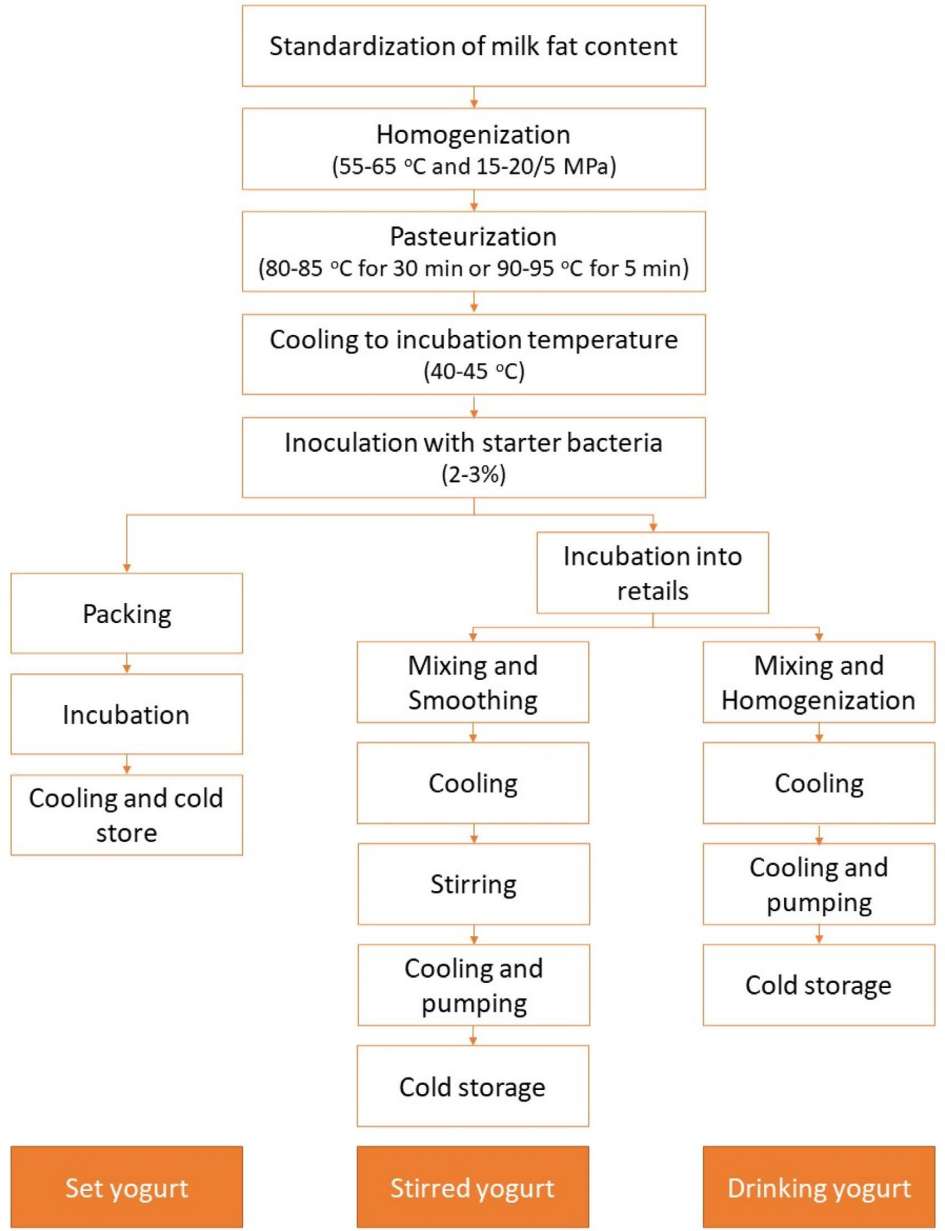
Flowchart illustrating yogurt production stages for set, stirred, and drinking yogurts.

Figure 2 shows the flow chart of the production technology of yogurts. Regardless of type—set, stirred, or drinking—the milk preparation begins with standardizing fat and dry matter content (Lee and Lucey 2010). This can be done through membrane filtration, vacuum evaporation, or adding milk powders (Robinson 2002). While effective, these methods may have downsides like higher energy use and potential impacts on the sensory qualities of yogurt. Ultrasound‐assisted filtration is a promising alternative, enhancing membrane efficiency (Sousa et al. 2016; Córdova et al. 2020; Soltani Firouz et al. 2023) and preserving nutritional content, which could improve yogurt texture and nutritional value (Nguyen and Anema 2017; Yuan et al. 2022). Additives such as stabilizers and vitamins may be added before heating (Chandan and O'Rell 2007; Teles and Flôres 2007). An increase in total solids (TS) content, resulting in enhanced firmness, $G^{’}$, complex viscosity (η*), and η (Krzeminski, Großhable, and Hinrichs 2011; Lucey 2002; Mistry and Hassan 1992; Penna, Converti, and Oliveira 2006a; Wu et al. 2009), which are crucial for consumer satisfaction.

Homogenization reduces fat globule size, improving yogurt texture and preventing creaming. Pasteurization follows homogenization to avoid lipolysis and ensure product quality. Homogenization is typically done at 15–20 MPa and 55°C–65°C (Chandan and O'Rell 2007), followed by heating the milk to 85°C–95°C for 30 min or 90°C–95°C for 5–10 min (Chandan and O'Rell 2007).

The milk is then cooled to 40°C–45°C or below 10°C if pretreatment exceeds packaging capacity. Starter cultures are added, and the milk ferments at 40°C–45°C for 2.5–3 h. If using frozen or concentrated cultures, fermentation takes 5–6 h (Corrieu and Béal 2016). Once the yogurt reaches a pH of 4.2–4.5, it is cooled below 10°C to stop acidification. Yogurt cooling can be achieved through two methods: one‐phase and two‐phase cooling (Tamime and Robinson 2007a). For plain set yogurt, a one‐phase cooling method is used, while stirred yogurt follows a two‐phase process, adding flavors and fruits before final cooling. The second cooling stage is conducted gradually within 12 h to enhance the quality of the yogurt. Rapid cooling can result in a weak gel structure and increase the risk of whey separation during storage (Özer 2009). Meanwhile, drinking yogurt is made by diluting stirred yogurt after fermentation. This process involves additional stirring and often incorporates a stabilizer to achieve a smoother, more liquid consistency (Sobhay, Hassan, and El‐Batawy 2019).

Finally, yogurt is packaged, with viscosity improving over 1–2 d as casein micelles hydrate. A 24–48 h delay in sale or distribution ensures proper stabilization (Shah 2003).

## 4 Types of Yogurt and Their Rheological Properties

The yogurt market boasts a diverse landscape, including more than a dozen distinct types, such as “sundae‐style” yogurt, Greek yogurt, Swiss‐style yogurt, whipped yogurt, and plant‐based yogurt. Each type further diversifies into sub‐categories based on a range of appealing flavors, sugar content, and fat content. This diversity reflects industry innovation and an ability to respond to consumer preferences, showcasing a deep understanding of varied tastes and nutritional needs.

Cup‐set yogurt production involves blending, pasteurization (optionally with homogenization), culturing, and pouring the yogurt mix into individual cups or containers before incubation. Once fermentation reaches a predetermined level, the cups are cooled. French yogurt exemplifies this type, typically featuring flavors but omitting pieces of fruit. Another variation is fruit on the bottom, or “sundae‐style” yogurt, where a pre‐prepared fruit layer rests at the base of the cup. The yogurt mix is gently deposited atop the fruit layer after incubation, resulting in a layered texture and unique flavor experience (Shah 2003). Cup‐set yogurt has a distinctive gel‐like texture that feels firm and holds its shape well (Jaros, Heidig, and Rohm 2007). The absence of post‐fermentation stirring helps maintain the integrity of the gel network, contributing to minimal whey separation and a thicker, creamier yogurt experience (Körzendörfer et al. 2018). Frozen yogurt combines the properties of traditional yogurt with the characteristics of ice cream. It is fermented and then frozen, creating a dessert with a tangy flavor and creamy texture (Rezaei et al. 2014).

High‐protein yogurt has gained popularity as a functional dairy product due to its improved nutritional profile and rheological properties. The high protein content results in a higher viscosity and $G^{\prime }$, indicating stronger elastic properties that enhance the firmness of the yogurt (Jørgensen et al. 2019). This can be achieved through two main methods: pre‐fermentation methods, which include adding milk powder, membrane filtration or evaporation, and post‐fermentation techniques, such as mechanical separation, membrane filtration, or straining (O'Rell and Chandan 2013). Greek yogurt, a high‐protein set yogurt, is produced by centrifuging or straining plain yogurt curd to remove whey, resulting in a product with approximately double the protein content of conventional yogurt. The straining process significantly increases viscosity, giving Greek yogurt its characteristic thick texture (Gyawali et al. 2022). Dairy solids may be incorporated to further enhance protein content and texture (Chandan 2017). The rise in popularity of Greek yogurt reflects consumers' increasing demand for nutrient‐dense, high‐protein dairy products that align with health and wellness trends.

Unlike traditional cup‐set yogurt, stirred or Swiss‐style yogurt undergoes bulk fermentation in tanks, followed by a post‐fermentation blending stage where flavorings, colorings, and fruits are carefully integrated (Shah 2003). This process gives the yogurt its characteristic smooth texture. The resulting mixture is then poured into individual cups, covered, and cooled. Compared to cup‐set yogurt, stirred yogurt typically exhibits a smoother texture and lower firmness and viscosity due to the disruption of the gel network during stirring (Mokoonlall, Nöbel, and Hinrichs 2016). The stirring process breaks down the gel structure, resulting in a distinct mouthfeel. Although stirring initially reduces viscosity, it can increase again during storage (Sodini et al. 2004). Australian yogurt, a rising star in the market, shares the characteristics of stirred yogurt but uses whole milk in the production process, contributing to a more pronounced flavor (Hersh 2021). Custard‐style yogurt stands out from stirred yogurt by using hydrocolloid thickeners that enhance viscosity and firmness, evoking the smooth texture of classic custard (Chandan 2017). Cream‐top yogurt celebrates the inherent separation of cream in unhomogenized yogurt mixtures. During storage, the lighter cream naturally rises, creating a distinctive two‐layer structure with a creamy top contrasting the yogurt body (Chandan 2017).

To produce drinkable yogurt, the fermented curd undergoes a homogenization process at a higher shear rate to achieve a smoother, lower‐viscosity consistency than traditional stirred yogurt. Colorings and flavorings can be added before or after homogenization. Ultra‐pasteurization and aseptic packaging are essential for shelf‐stable yogurt products (Chandan 2017).

Whipped yogurt, also known as yogurt mousse, achieves its light and airy texture through gentle aeration, in which air bubbles are introduced into the yogurt mixture, creating a mousse‐like consistency (O'Rell and Chandan 2013). To maintain this structure, stabilizers such as gelatin, carrageenan, and xanthan gum increase viscosity, while emulsifiers such as mono‐ and diglycerides aid in bubble formation by reducing surface tension (O'Rell and Chandan 2013). Nitrogen is often used in the aeration process to create fine gas bubbles, resulting in a stable foam structure. Compared to traditional yogurt, added sugars and stabilizers enhance the texture and stability of the product (Tamime and Robinson 2007b).

Plant‐based yogurt is becoming increasingly popular as an alternative to traditional yogurt, due to dietary preferences, lactose intolerance, and environmental concerns (Jaeger et al. 2023). Made from sources like soybean, coconut, almond, and walnut, these yogurts are formulated to mimic the appearance and creamy texture of traditional yogurt (Grasso, Alonso‐Miravalles, and O'Mahony 2020). However, acidification in these plant‐based systems can destabilize proteins, resulting in weak, discontinuous gel structures and increased serum separation (Bernat et al. 2014). To address this, gelling agents such as agar, pectin, starches, and xanthan gum are often used to stabilize the particles in the suspension, promote structure formation, and better reproduce the desired viscosity and mouthfeel of yogurt (Grasso, Alonso‐Miravalles, and O'Mahony 2020).

## 5 Influencing Factors on the Rheology of Yogurt

### 5.1 Milk Origin

Cow milk is the primary ingredient in commercial yogurt production. However, alternative milk sources such as sheep, buffalo, camel, and goat milk are also viable options (Wang et al. 2025). The yogurt industry has been experimenting with milk blends as well. The texture and rheological properties of yogurt are influenced by the total protein and TS content of the milk (Terzioğlu et al. 2023). Typically, skimmed milk (SM) powder plays a crucial role in yogurt production by increasing the TS content of the milk base, ensuring the desired texture and consistency in the final product (Karam et al. 2013). Understanding the impact of milk composition on yogurt properties is essential for achieving consistent quality and meeting consumer expectations.

The impact of milk origin on yogurt rheology through gelation has been evaluated for different types of milk, including cow (Tamime, Kalab, and Davies 1989), goat (Bruzantin et al. 2016), sheep (Mohameed, Abu‐Jdayil, and Al‐Shawabkeh 2004), and camel milk (Sobti et al. 2020). Sheep milk has the highest viscosity, followed by goat, cow, and camel milk (Jumah, Shaker, and Abu‐Jdayil 2001). This variation in viscosity highlights the diverse rheological properties of yogurt, offering opportunities to differentiate products based on milk choice. The structural development of curd during acid gelation occurs in three stages: An induction stage with no noticeable change in viscosity, a flocculation phase marked by a significant increase in viscosity, and a final stage with reduced viscosity due to the rearrangement, contraction, and aggregation of casein micelles.

In the case of cow, sheep, and goat milk, distinct transient viscosity phases can be characterized by mathematical equations. However, camel milk exhibits minimal viscosity changes during gelation. Using a power law model, the flow characteristics and thickening properties of curds from various milk origins can be quantified (Jumah, Shaker, and Abu‐Jdayil 2001). These findings suggest that yogurt exhibits shear‐thinning properties during gelation. Notably, goat milk yogurt has suboptimal textural quality and a fragile gel structure due to variations in protein composition, especially αs1‐casein content, compared to cow milk (Park et al. 2007; Domagała 2009). In contrast, sheep milk has a higher TS content than goat and cow milk, resulting in a more desirable yogurt texture. To enhance the gel network texture, whey protein can be added to goat milk yogurt (Gursel et al. 2016).

Mudgil et al. (2018) demonstrated that adding gelatin to camel milk significantly enhanced its rheological characteristics, making the yogurt almost comparable to commercial cow milk yogurt. This improvement was accompanied by a transformation from a watery to a gel‐like structure, as indicated by the increased $\eta^{*}$ at gelatin levels above 0.50%. Additionally, gelatin effectively decreased syneresis, or the separation of whey from the yogurt gel, particularly at gelatin concentrations of 1% and 1.25%. This demonstrates the potential of additive‐based solutions to overcome the inherent limitations of certain milk types in yogurt production, reflecting a pragmatic approach to product innovation where the strategic use of additives aligns with consumer expectations of quality and consistency.

### 5.2 Non‐Thermal Treatment

Common heat treatments of milk, such as pasteurization or sterilization, can reduce nutritional content and create off‐flavors that affect sensory quality. To mitigate these issues, alternative non‐thermal processing technologies, such as ultrasound (Sfakianakis, Topakas, and Tzia 2015; Gursoy et al. 2016; Körzendörfer et al. 2019; Delgado et al. 2020; Yuan et al. 2022; Akdeniz 2023), microfluidization (Cobos, Horne, and Muir 1995; Augustin, Sanguansri, and Htoon 2008; Ciron et al. 2010, 2011), HPP (Johnston and Murphy 1994; Penna, Gurram, and Barbosa‐Cánovas 2006b; Ramaswamy, Chen, and Rattan 2015), and microfiltration (Debon, Prudêncio, and Cunha Petrus 2010; Debon et al. 2012; Rinaldoni, Campderrós, and Pérez Padilla 2012; Bong and Moraru 2014; Jørgensen et al. 2017; Tian et al. 2022), have been widely used.

In a study by Korzendorfer et al. (2019), SM underwent acidification at 43.5°C and was then sonicated using a sonotrode operating at 20 kHz and 20 W, with a 1 s pulse cycle (0.2 s on, 0.8 s off) to maintain temperature stability. Sonication was applied until pH reached 5.1, resulting in smoother samples with some large aggregates and more compact microgel particles. This led to a decrease in viscosity, gel firmness, and adhesive structure.

Meanwhile, Jørgensen et al. (2017) studied the impact of casein micelle size on the rheology of low‐fat set yogurt with high protein content (5.6% crude protein). They used microfiltration (0.20 μm membranes) to separate SM into two fractions: “small” (∼129 nm) and “large” (∼183 nm) casein micelles. The permeate enriched with small casein micelles underwent further concentration with 0.10 μm membranes. Yogurt milk bases were then heat‐treated at 95°C or 75°C for 5 min. Yogurt made with small casein micelles had a higher $G^{\prime }$ and increased firmness compared to those with large casein micelles, attributed to the higher concentration of κ‐casein in yogurt with small casein micelles. These results highlight the potential of ultrasound and microfiltration in producing yogurt to meet consumer demand. Future research should explore the link between casein micelle size distribution and consumer perception of texture in stirred and set yogurts.

**FIGURE 3:**
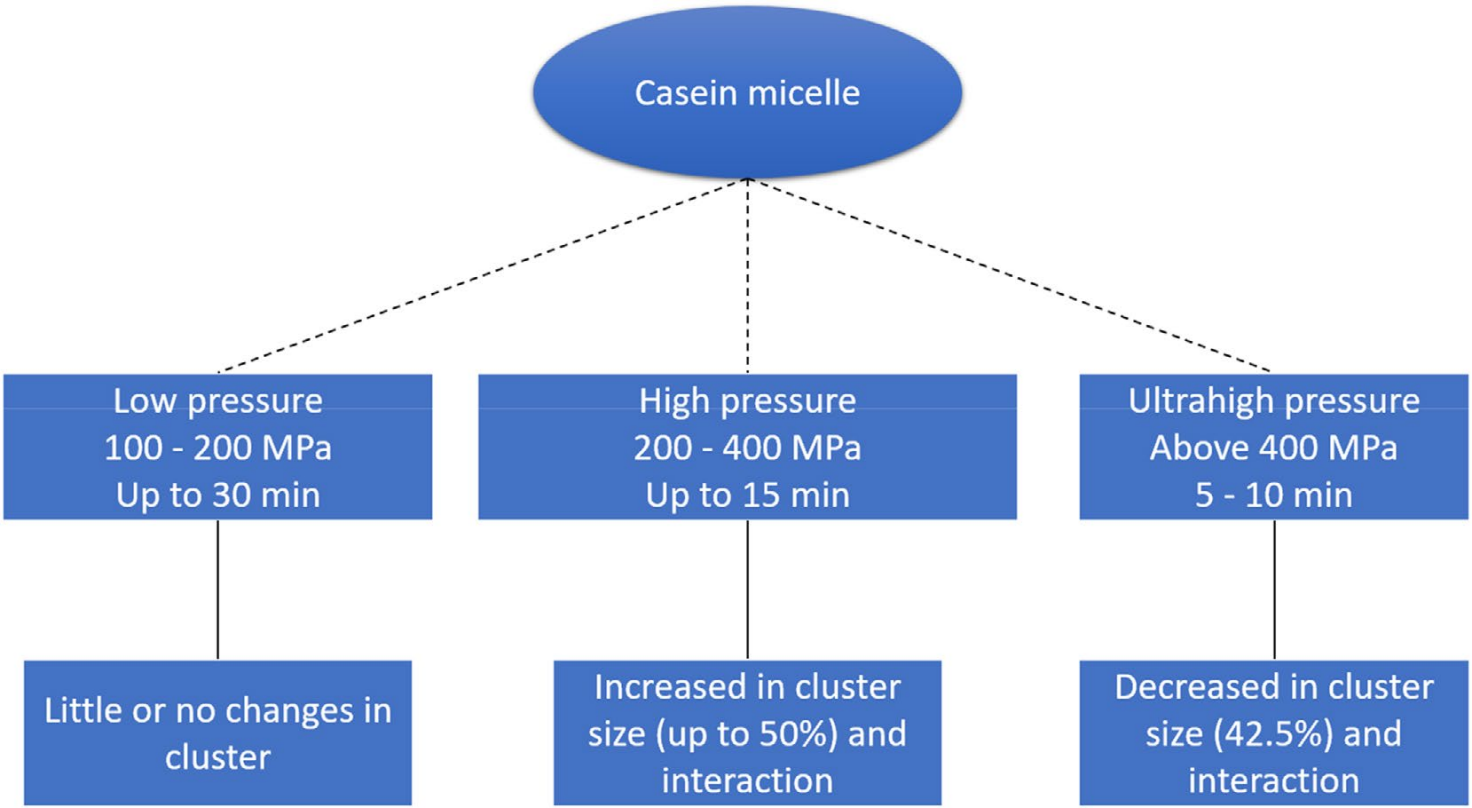
Effects of HPP on casein micelle.

HPP breaks down large, complex micelles into smaller, simpler structures. The effect of pressure on casein micelles is shown in Figure 3. These smaller micelles are more mobile and can interact more easily, facilitating the creation of a stronger, more stable gel network (Johnston and Murphy 1994). The SM is typically subjected to short‐term high‐pressure treatment like 676 MPa for 5 min (Penna, Gurram, and Barbosa‐Cánovas 2006b), or a specific pressure profile in the range of 100–600 MPa with processing time up to 1 h (Johnston and Murphy 1994). This technique increased consistency, creaminess, and thickness in yogurt, eliminating the need for additional stabilizers. Moreover, the viscosity of yogurts increased with increasing pressure in the range of 100–600 MPa and treatment time. Notably, HPP significantly increased the $G^{\prime }$ of set and stirred yogurt. The use of pressure‐treated milk led to distinct strain, frequency‐dependent, and gel properties in the yogurt samples. This method not only improves product quality but also simplifies the production process, providing a more sustainable method of producing yogurt.

Similarly, Ciron et al. (2011) investigated the effects of microfluidization (150 MPa) on the rheology of reduced‐fat yogurt. Microfluidization significantly improved desirable texture and creaminess properties, resulting in higher viscosity, $\tau_{\text{yield}}$, and hysteresis effect.

Ultraviolet (UV) light has also emerged as a promising non‐thermal, non‐chemical decontamination technique in liquid food processing (Koutchma 2009). Unlike HPP and microfluidization, which primarily target texture, UV‐C processing enhances yogurt safety by reducing microbial load. However, the application of short‐wave UV‐C light for decontamination has previously been limited to transparent liquids and beverages because its effectiveness is reduced in opaque systems due to the large amounts of UV‐C absorbed and dispersed by suspended particles (Koutchma 2009). To overcome this limitation, Vásquez‐Mazo et al. (2019) processed milk using short‐wave UV‐C light (1060 mJ/cm^2^) combined with thermal treatment (85°C) under vacuum conditions (400 mmHg). They found that UV‐C‐treated yogurts exhibited significantly reduced syneresis (60% lower) and the highest white index among all yogurt samples.

Each of these technologies offers a way to overcome the limitations of traditional heat treatments, creating opportunities for the dairy industry to produce yogurt with improved sensory, textural, and nutritional qualities. By carefully selecting and applying these techniques, manufacturers can create dairy products that meet consumer demands for healthfulness, taste, and texture.

### 5.3 Homogenization

Before producing yogurt, milk must be homogenized at a pressure of 10–20 MPa and a temperature of 55°C–65°C, typically less than 50 μs, as milk passes through the homogenizer (Chandan and O'Rell 2007). This process significantly reduces the size of fat droplets, resulting in an expanded surface area while maintaining the integrity of the casein micelles. Smaller fat globules, covered by absorbable casein and denatured whey proteins, are incorporated into the gel network during acid‐induced coagulation (Sameen et al. 2010). This integration offers several advantages in yogurt production, such as better dispersion of fat globules, enhanced gel firmness, reduced whey separation, and improved texture (Tamime and Robinson 2007a).

Nguyen et al. (2015) studied the effect of homogenizing buffalo milk on the rheological properties of yogurt at pressures of 8 and 16 MPa. Their study revealed significant variations in the microscopic structure of buffalo yogurt produced from homogenized milk, characterized by a densely bound protein matrix containing smaller embedded fat globules. These structural changes led to a notable increase in the flow characteristic, gel firmness, and $G^{\prime }$ of yogurt. This phenomenon is thought to be related to differences in the specific surface area and size of the fat globules, as well as changes in the composition of fat globule membranes in milk. The protein layer surrounding the homogenized fat globules enhances structural integrity and actively participates in network formation during fermentation (Xiong and Kinsella 1991).

The impact of homogenization pressure, specifically at 8 and 16 MPa, on the rheology of buffalo milk yogurt was significant (Nguyen et al. 2015). Homogenization at 8 MPa led to an increase of over 80% in the $G^{\prime }$ upon finalizing yogurt fermentation compared to the control sample. While buffalo yogurt made from milk homogenized at 16 MPa exhibited a higher $G^{\prime }$ than the control sample, it remained lower than the yogurt homogenized at 8 MPa upon finalizing fermentation. This result deviates from previous research on rennet and acid gels produced from cow milk (Michalski et al. 2002) and is likely attributed to the distinct characteristics of buffalo milk, such as larger fat globules and higher fat content. Homogenization enhanced the stability of the yogurt network, making it less susceptible to deformation, as evidenced by lower phase angle values, reflecting the ability network to resist deformation. Interestingly, no statistically significant difference in tanδ values and syneresis levels was observed between yogurt samples processed with the two homogenization pressures. Moreover, homogenized buffalo yogurt demonstrated a substantial reduction in syneresis, with a sixfold decrease compared to non‐homogenized yogurt (Nguyen et al. 2015). No statistically significant difference in syneresis was observed between yogurts homogenized at different pressures. Considering these results, a lower homogenization pressure of 8 MPa was recommended to effectively minimize whey separation in buffalo yogurt production. The reduction in syneresis is tentatively attributed to the reduced size of fat droplets and the formation of a dynamic protein membrane, resulting in a gel matrix with increased cross‐linking in homogenized yogurts.

The impact of conventional homogenization and microfluidization at 20 and 150 MPa, respectively, was compared on the rheological behavior of low‐fat (1.5%) and non‐fat (0.1%) stirred yogurts (Ciron et al. 2010). The study demonstrated that both homogenization methods produced viscoelastic yogurts independent of fat content, with overlapping $G^{\prime }$ and $G^{"}$ values. While homogenization had a negligible effect on viscoelastic characteristics, both moduli values of non‐fat yogurt produced from microfluidized heat‐treated milk were slightly lower than those of homogenized yogurt. Microfluidized milk consistently produced yogurt with a greater hysteresis loop area and $\tau_{\text{yield}}$ compared to conventionally homogenized milk, a difference particularly pronounced in low‐fat yogurt.

Jiao et al. (2023) investigated the high‐pressure homogenization of the microstructure and rheology of walnut yogurts. They found that homogenization markedly improved the rheological properties of walnut yogurt, with significant increases in $G^{\prime }$ and $G^{"}$ values, indicating enhanced gel strength and elasticity. Homogenization‐induced particle size reduction contributed to improved water‐holding capacity and enhanced texture attributes, including firmness and consistency. Confocal laser scanning microscopy revealed that homogenization resulted in a reduction in fat and protein micelle size, leading to a more compact gel network structure. Identifying suitable high‐temperature roasting parameters for fermented walnut milk processing was proposed as necessary.

### 5.4 Starter Cultures

The selection of starter culture significantly influences the rheological properties of yogurt, affecting the texture, firmness and stability of the final yogurt product (Bouzar, Cerning, and Desmazeaud 1997). The strains of *S. thermophilus* and *L. delbrueckii* subsp. *bulgaricus* are primarily utilized in yogurt production due to their synergistic interaction and positive effect on yogurt characteristics (Das, Choudhary, and Thompson‐Witrick 2019). In particular, exopolysaccharides (EPS)‐producing bacterial strains contribute to the viscoelastic properties of yogurt by enhancing gel strength and reducing syneresis. The amount of produced EPS can vary significantly depending on the lactic acid bacteria used in starters, influencing the yogurt firmness, viscosity, and stability. For example, *S. thermophilus* can produce EPS at concentrations between 50 and 350 mg/L, while *L. delbrueckii* subsp. *bulgaricus* typically produces EPS in the range of 60–150 mg/L, contributing to a softer texture and reduced viscosity (Marshall 1987; Gruter et al. 1993).

Prasanna, Grandison, and Charalampopoulos (2013) demonstrated that using EPS‐producing *Bifidobacterium longum* subsp. *infantis* enhanced yogurt firmness, texture and $G^{\prime }$ values within 28 d of storage at 4°C, indicating the protein reorientation within the gel network and the strengthening of EPS‐protein and protein–protein interactions. A study by Ramchandran and Shah (2009) examined the influence of *S. thermophilus* cultures, which produce or do not produce EPS, on the textural and rheological characteristics of inulin‐supplemented yogurt within a storage period of 28 d at 4°C. Their findings revealed that yogurt firmness did not change significantly during storage. However, non‐EPS‐producing yogurts consistently exhibited higher firmness, $G^{\prime }$, and $G^{"}$ compared to EPS‐containing yogurts. They proposed that interference of inulin with casein micelle interactions could lead to weaker gels in EPS‐containing yogurt. The empty spaces surrounding EPS‐producing strains might further weaken yogurt structures. During storage, all yogurts demonstrated $G^{\prime }>G^{"}$, confirming an unstable gel microstructure. Hess, Roberts, and Ziegler (1997) compared yogurt properties with three EPS‐producing variants of *L. delbrueckii* subsp. *bulgaricus* and a non‐EPS‐producing one. EPS‐producing yogurts required less force to penetrate and showed a gradual decrease in $G^{\prime }$ and $G^{"}$ moduli between 5% and 10% strain, with a shift in slope beyond 10% strain. In contrast, non‐EPS yogurts showed a continuous reduction in $G^{\prime }$ and $G^{"}$ as non‐producing strain content rose above 5%. These findings suggest that EPS in yogurt protects the casein network, helping to prevent shear‐induced disruption at various shearing rates.

Regarding the impact of “ropy” EPS‐producing strains, Rawson and Marshall (1997) investigated the effect of ropiness on the rheological properties of stirred yogurt using non‐ropy ( *S. thermophilus* ) and ropy ( *L. delbrueckii* subsp. *bulgaricus*) strains. The presence of ropy bacteria strains significantly increased the viscosity of yogurt compared to yogurts produced using non‐ropy cultures. Khanal and Lucey (2017) compared the production of EPS and the resulting gel properties of yogurts fermented by two *S. thermophilus* strains, St‐143 and ST‐10255y. They found that the two strains produced significantly different amounts and types of EPS during fermentation. Strain St‐143 produced higher amounts of EPS earlier in the fermentation process, leading to weaker gels with higher maximum loss tangent values. This phenomenon was attributed to discrepancies in the molecular masses and chemical structures of the EPS produced by both strains. The findings of this study highlighted the importance of careful strain selection for producing yogurt with desired gel properties.

### 5.5 Fat Content

Milk fat significantly influences the sensory properties of dairy products, affecting flavor, texture, appearance, mouthfeel, consistency, porosity, and lubricity (Lee and Lucey 2006; Cayot et al. 2008). In milk, fat globules are immobilized by the casein network, appearing as smooth, prominent spheres within the protein gel structure (Keogh and O'Kennedy 1998). Milk proteins naturally attach to the surface of these fat globules, a crucial interaction for forming the structural network of yogurt (Sandoval‐Castilla et al. 2004). Additionally, fat globules and unbound, coiled whey proteins dispersed in the serum form the remaining hydrosoluble fraction.

Consumers are increasingly inclined toward healthier dairy products, favoring options with reduced fat content. Yogurt is primarily categorized into three groups based on fat content: high‐fat, low‐fat, and fat‐free. According to USDA guidelines (USDA 2001), non‐fat yogurt should have less than 0.5% fat, low‐fat yogurt should have between 0.5% and 2% fat, and high‐fat yogurt should have above 3% fat, with the milk solid not‐fat compounds remaining at 8.25%. For decades, the U.S. yogurt market has been dominated by low‐fat and fat‐free varieties, reflecting the widespread belief that minimizing fat intake is essential for a healthy diet. Lite or light yogurt offers a lower‐calorie option, containing one‐third fewer calories or 50% less fat than regular yogurt. These yogurts are generally sugarless and sweetened with intense, non‐nutritive sweeteners. However, low‐fat yogurts often have sensory and textural shortcomings. Reducing fat content can lead to reduced viscosity due to lower TS content, resulting in undesirable texture and mouthfeel (Aziznia et al. 2008). Conversely, Laiho et al. (2017) reported that skim yogurts exhibited increased firmness and viscosity compared to their full‐fat counterparts. Incorporating fat replacers can alter the texture of reduced‐fat yogurts. Various dairy ingredients, such as skimmed milk powder (SMP), whey protein concentrate (WPC), and whey protein isolate (WPI), are often included in yogurt formulations for their favorable nutritional profile and functional properties (De Wit 1998; Hau and Bovetto 2001).

Torres et al. (2018) conducted a study examining the effects of ten microparticulated whey protein (MWP) preparations with various physical and chemical properties on the microstructure and rheological properties of low‐fat stirred yogurt (0.5% w/w fat) at two different levels of total protein (4.25% and 5.0% w/w). They found that η reached a maximum at a particular shear rate for all yogurts, coinciding with $\tau_{\text{yield}}$. All yogurts exhibited flow behavior at shear rates exceeding this value, as indicated by decreasing viscosity values with increasing shear rates, confirming the shear‐thinning pattern. Adding more whey protein microparticles significantly increased yogurt viscosity, with a few notable exceptions. A weak gel‐like property of yogurt was discovered by the frequency sweep. The presence of fat significantly impacted yogurt gel strength, with higher fat content associated with stronger gels and higher $G^{\prime }$. Conversely, eliminating fat led to a weaker gel structure. In some yogurt formulations, replacing fat with MWP improved gel strength, especially when the protein concentration increased to 5.0% (w/w). However, the effectiveness of this substitution depends on the types of microparticulated components used. The significant impact of MWP on yogurt viscosity suggests their potential as texture‐modifying agents, offering opportunities for tailored yogurt formulations to meet specific consumer preferences. On the other hand, Xu et al. (2008) studied the effects of fat content (0%, 1.5%, and 3.5%) and heat pretreatment on the structure formation of set yogurt. This study stated that higher fat content and heating temperature accelerated the gelation process and produced a rigid gel structure.

Milk is typically homogenized at pressures of 15–20 MPa and temperatures of approximately 65°C–70°C, using a single‐ or two‐stage process (Chandan and O'Rell 2007). This high‐pressure treatment reduces fat globule size and partially coats their surfaces with proteins, enhancing their hydrophilicity. Consequently, these smaller, protein‐coated fat globules integrate more effectively into the yogurt gel network, improving firmness and mouthfeel but slightly reducing homogeneity. Alternatively, ultra‐high‐pressure homogenization at 200–300 MPa can be applied, further increasing firmness and water‐holding capacity of yogurt. This intense process denatures whey proteins, allowing them to adhere to casein micelles and fat globules, thereby strengthening the gel structure. Notably, unhomogenized milk with larger fat globules can disrupt the gel network, diminishing consistency and adversely impacting overall yogurt quality.

### 5.6 Fat Replacers

Researchers have introduced fat replacers to restore the textural and structural properties of low‐fat yogurt and enhance its overall quality. These fat replacers help strengthen the yogurt gel network, resulting in desirable rheology, texture, and sensory characteristics such as smoothness, mouthfeel, firmness, and creaminess. Common additives in low‐fat yogurt production include whey proteins, fat replacers, and thickeners to achieve optimal quality (Haque, Richardson, and Morris 2001; Haug and Hvam 2007; Damin et al. 2009). In addition to proteins, various additives like gelatin, chitosan, modified starch, and inulin have been explored to enhance texture by increasing the TS content.

Extensive testing of some hydrocolloids, including modified starch (0.5%–1.5%), xanthan gum (0.005%–0.015%), gelatin (0.5%–1.5%), and carrageenan (0.01%–0.08%), has been conducted to assess their impact on the rheological properties of low‐fat (0.1%) yogurt (Nguyen et al. 2017). At a shear rate of 50 s^−1^, the viscosity ($\eta_{50}$) remained stable across different fat levels for all yogurt samples. However, carrageenan and xanthan gum significantly increased $\eta_{50}$ at low concentrations. In contrast, increasing gelatin content led to a decrease in $\eta_{50}$ for low‐fat yogurt. $G^{\prime }$ and $G^{"}$ values in low‐fat yogurt decreased as gelatin content increased, with the decrease becoming more pronounced at higher gelatin levels. Adding 1.5% gelatin to skim milk (SM) yogurt decreased $G^{\prime }$ and $G^{"}$ values by an order of magnitude compared to the control skim sample. Conversely, carrageenan and xanthan gum interacted with milk proteins in the yogurt gel network, leading to a significant increase in $G^{\prime }$ and $G^{"}$.

A summary of the effects of using fat replacers on the microstructural and rheological properties of yogurt is provided in Table 1 and Table 2 according to the substituting compound: carbohydrates (Part A), and whey protein and derivatives (Part B).

**TABLE 1:** Influence of fat replacers on the microstructural and rheological properties of yogurt. Part A: Carbohydrates.

Ref.	Product	Fat replacer	Positive effects	Negative effects
Costa et al. 2022	Low‐fat goat milk yogurts	Inulin, maltodextrin (5%)	Enhanced the η and firmness of goat milk yogurt Improved the gel structure formation	—
Lobato‐Calleros et al. 2014	Reduced‐fat stirred yogurt	Starch from tapioca, waxy maize, and native maize (1%)	Creation of a more durable and more uniformly distributed acidified gel base Reduced syneresis in reduced‐fat yogurt Increased η—shear rate profiles, higher dynamic viscoelastic moduli	Minimal changes in their viscoelastic and flow behavior.
Lin et al. 2022	Low‐fat yogurt	Tremella fuciformis polysaccharides (TFP) (0.025%–0.100%)	Enhanced the adhesion, firmness, cohesion, and viscosity Filled the porous protein network, reinforcing the three‐dimensional structure of yogurt	—
Arango, Trujillo, and Castillo 2020	Set yogurt	Inulin (1.6% and 3.2%)	Increased $G^{’}$ and gel firmness Accelerated the rate of acidification	Anticipated the beginning of milk coagulation Increased spontaneous syneresis with no effect on water holding capacity
Sharma et al. 2018	Reduced‐fat yogurt	Octenyl succinylated pearl millet ( *Pennisetum typhoides* ) starch (0.5%–2.0%)	Increased firmness, flow point, $G^{’}$, and $G^{”}$ Reduced syneresis	—
Santiago‐García et al. 2021	Fat reduced yogurt	Fructans (agavins) from *Agave potatorum* and *Agave angustifolia* (3% and 6%)	Increased viscosity fivefold Reduced syneresis Improved mounthfeel with expanded the linear viscoelastic range	—

**TABLE 2:** Influence of fat replacers on the microstructural and rheological properties of yogurt. Part B: Whey protein and derivatives.

Ref.	Product	Fat replacer	Positive effects	Negative effects
Zhang, Mccarthy, Wang, Liu, and Guo, 2015	Non‐fat goats' milk yogurt	Heat‐treated whey protein concentrate (1.2%) and pectin (0.35%)	Stable viscosity of yogurt during storage Greater viscosity and reduced syneresis More cohesive protein matrix along with casein micelles, ultimately enhancing both texture and water retention	—
Li et al. 2021	Low‐fat yogurt	Thermal‐denatured whey protein isolate‐milk fat emulsion gel microparticles (WPI‐EG) (0.5%–1.5%)	Increased elasticity, viscosity, G′ and G′′ Improved firmness and water‐holding capacity Reduced pore size in yogurt gel network	—
Fang et al. 2019	Low‐fat yogurt	Polymerized whey protein (PWP) (1.4%)	Firmer structure and higher viscosity; Formation of protein aggregates and increased concentration of protein polymers.	—
Hossain et al. 2020	Plain‐type yogurt and reduced‐fat yogurt (< 0.5 g/100 g)	Extruded MWP (4%)	Lower rates of syneresis and higher firmness and thickness and creaminess	Decreased viscosity
Godoy‐García et al. 2020	Sugar‐reduced Greek‐style yogurt	Glycomacro‐peptide (GMP) (0.25%, 0.50%, and 0.75%)	Reduced graininess and syneresis; Softer texture due to electrostatic bonds between the glycosylated chains and the casein chain of GMP.	—

Strategies such as incorporating inulin, maltodextrin, starch, or polysaccharides have shown promising results, leading to a firmer texture, increased viscosity, and reduced syneresis in yogurt. Innovations like WPI‐EG have significantly enhanced elasticity and water‐holding capacity of yogurt, further improving its overall structure. As the industry continues to explore new fat substitutes and additives, there is a significant opportunity to innovate and create low‐fat yogurt products that meet consumer demand for healthy options while providing an exceptional sensory experience. Additionally, integrating emerging technologies and scientific advances can support the development of more complex yogurt formulations, further enhancing taste, texture, and nutritional value.

### 5.7 Fortification With Fruits and Herbs

Incorporating fruit into yogurt helps diversify choices for consumers, as yogurts with added flavors and fruits are widely consumed. Commonly used fruits include strawberry (Lubbers et al. 2004), apple (Wang, Kristo, and LaPointe 2019), mango (Singh and Muthukumarappan 2008), cherry (Celik, Bakrc, and Şat 2006), and banana (Safdari et al. 2021), especially in commercial yogurt in the United States, where low‐fat yogurts with added fruit are very popular. Although research is ongoing into incorporating vegetables and herbs into yogurt, these ingredients are not yet widely available in the US, and concerns about shelf life have not been fully addressed (Clark, Michael, and Schmidt 2019). Addressing these concerns will be important to unlocking the full market potential of such innovative yogurts. This highlights the opportunity for manufacturers to capitalize on emerging consumer trends by addressing the logistical challenges associated with incorporating new ingredients.

A study on the rheology of yogurt enriched with herbal extracts such as marjoram, sage, hawthorn, and thistle at mass concentrations ranging from 0.25% to 1.0% was conducted by Dabija et al. (2018). All yogurt variants exhibited weak gel and thixotropic properties. Initially, yogurt without added herbs had a higher overall viscosity than yogurt with added herbs. However, by day 28, yogurt supplemented with herbs had a higher viscosity than yogurt without added herbs. In all cases, $G^{\prime }$ exceeded $G^{\prime \prime }$, and by day 28, the viscoelastic modulus value was higher in the herbal‐fortified yogurt, indicating a more durable gel network.

Similarly, Lubbers et al. (2004) observed an increase in the η and consistency index of stirred fruit yogurt, specially prepared with strawberry powder, during 28 d storage period at 10°C. During storage, both the η and the consistency index increased with no noticeable change in the flow characteristic index due to fruit preparation. Changes in the rheological properties of yogurt during storage were attributed to the generation of lactic acid and EPS through continuous microorganism activity, strengthening the protein network.

Overall, the addition of fruits and herbs to yogurt may be a promising avenue for product development that meets consumer preferences for nutritious, flavorful, and diverse yogurts. However, relevant issues need to be addressed to gain consumer acceptance, especially for less conventional dietary supplements such as vegetables and herbs.

The influence of these factors on the rheological properties of yogurt is shown in Table 3, Table 4 and Table 5 according to the factor: milk origin and starter culture (Part A), applied treatment during processing (Part B), and compounds including fortification (Part C).

**TABLE 3:** Summary of results and challenges of factors on the rheological properties of yogurt. Part A: Milk and starter cultures.

Factor	Source	Method	Results	Challenge
Milk origin	Dairy	Addition of SMP, whey proteins, gelatin	Sheep milk: highest viscosity and desirable texture due to higher TS content (Park et al. 2007) Goat milk: fragile gel structures; improvements with whey protein addition (Gursel et al. 2016) Camel milk: no gel formation, improved texture and rheological properties with the addition of gelatin, especially at 1% and 1.25% levels (Mudgil et al. 2018)	Goat milk: requires formulation adjustments to improve texture, finding the optimal casein: whey protein ratios Camel milk: Modification of camel milk yogurt with the addition of gelatin provided a solution to previous limitations; however, sensory analysis showed lower scores in all parameters except texture compared to yogurt cow milk
Starter cultures	Dairy	EPS‐producing and non‐producing strains	EPS‐producing strains: improved viscosity and texture (Marshall 1987; Gruter et al. 1993) Synergistic cultures of *S. thermophilus* and *L. delbrueckii* subsp. *bulgaricus* enhanced firmness and gel properties (Hutkins 2007). *Bifidobacterium longum* ssp. *infantis* increased yogurt firmness over time, (Prasanna, Grandison, and Charalampopoulos 2013) Non‐EPS‐producing strains: higher firmness due to interference of inulin (Ramchandran and Shah 2009)	Optimizing strain selection to balance texture, flavor and functional benefits Understanding synergistic behavior across cultures, and their interactions with other yogurt ingredients

**TABLE 4:** Summary of results and challenges of factors on the rheological properties of yogurt. Part B: Treatments.

Factor	Source	Method	Results	Challenge
Non‐thermal treatment	Dairy	Ultrasound, microfluidization, HPP, microfiltration, UV‐C	Ultrasound and microfiltration: Enhanced smoother consistency (Jørgensen et al. 2017; Körzendörfer et al. 2019) HPP: improved gel network strength and stability and increased viscosity and creaminess without additional stabilizers (Penna, Gurram, and Barbosa‐Cánovas 2006b) Microfluidization enhanced texture, creaminess, and viscosity, indicating a more desirable structure (Ciron et al. 2011) UV‐C combined with thermal treatment under vacuum: reduced syneresis and improved whiteness (Vásquez‐Mazo et al. 2019)	Each technology requires specific equipment and optimization Compatibility of UV‐C with the physical characteristics of food products, design of UV reactors, and ensuring sufficient UV dosage are challenges Balancing the benefits of non‐thermal treatments with cost, scalability, and consumer acceptance
Homogenization	Dairy	Standard (10–20 MPa) f—Comparison at 20 and 150 MPa for low‐fat and non‐fat yogurts	Enhanced gel firmness, reduced whey separation, and improved texture (Tamime and Robinson 2007a). For buffalo milk, 8 MPa homogenization is superior to 16 MPa in reducing whey separation and improving gel structure (Nguyen et al. 2015).	Optimizing the homogenization process Balance texture improvements with minimized whey separation and maintaining overall quality throughout the shelf life
	Plant‐based	High‐pressure homogenization for walnut yogurts	High‐pressure homogenization significantly improved gel strength and elasticity in walnut yogurt (Jiao et al. 2023).	Determining suitable high‐temperature roasting parameters for fermented walnut milk processing

**TABLE 5:** Summary of results and challenges of factors on the rheological properties of yogurt. Part C: Compounds.

Factor	Source	Method	Results	Challenge
Fat content	Dairy	High‐fat, low‐fat, and fat‐free variations	High fat: stronger gels and higher viscosity due to the immobilization of fat globules by the casein network, enhancing mouthfeel and texture (Keogh and O'Kennedy 1998). Low‐fat and fat‐free: reduced viscosity and weaker gel structures, leading to less desirable texture and mouthfeel (Aziznia et al. 2008)	Low‐fat yogurts need additional ingredient to improve the sensory properties and meet consumer expectation (e.g., SMP, WPC, WPI)
Fat replacers	Dairy	Various fat replacers (inulin, maltodextrin, starch, gelatin, chitosan, modified starch, and others)	Improved texture, viscosity, gel firmness, smoothness, mouthfeel, and creaminess (Haque, Richardson, and Morris 2001; Haug and Hvam 2007; Damin et al. 2009)	Determining optimal concentration and type of fat replacer for desired texture and sensory attributes and ensuring compatibility of fat replacers with the yogurt matrix
Fortification with fruits and herbs	Dairy	Incorporation of fruits and herbs	Enhanced viscosity and consistency, the gel network strength over time giving weak gel and thixotropic properties to yogurt enriched with herbal extracts (Lubbers et al. 2004; Dabija et al. 2018).	Shelf‐life concerns Addressing consumer acceptance of these new yogurts Maintaining the quality of natural ingredients in yogurt matrix

## 6 Methodologies for Assessing Yogurt Rheology

The choice of instrument for measuring rheological properties of yogurt depends on two key factors: the yogurt characteristics and the specific information desired from the measurement. These parameters can be determined via various rheological tests, including viscometry, amplitude sweep, frequency sweep, time sweep tests, back extrusion test, and texture profile analysis (TPA).

Standardized cylinder‐cup viscometers have inherent limitations in comprehensively characterizing the flow of yogurt. These devices typically measure viscosity at a pre‐selected rotational speed, giving an apparent viscosity that does not reflect the true properties of the yogurt under different shear conditions. Furthermore, failing to capture all yogurt shear rates can lead to a fragmented understanding of its flow characteristics, potentially resulting in misinterpretations of its processing behavior and sensory attributes (Whaley, Templeton, and Anvari 2019).

Rheological measurements can provide comprehensive information about the structure of yogurt. However, an important aspect of rheological analysis is the preservation of the fine structure of the sample during preparation and loading. This can be especially challenging with set yogurt (Karagül‐Yüceer and Drake 2013; Whaley, Templeton, and Anvari 2019). Removing set yogurt from the container often disrupts the gel network formed by the complex interaction of casein chains and whey protein aggregates, creating a three‐dimensional structure with both granular and heterogeneous properties (Laiho et al. 2017). Despite this challenge, researchers have developed various rheological methods to characterize the structure‐related properties of yogurt samples.

Oscillatory rheometry, in particular, characterizes both the gelation process and the subsequent behavior of the established gel network (Boubellouta, Galtier, and Dufour 2011). There are two types: small amplitude oscillatory rheology (SAOR) and large amplitude oscillatory rheology (LAOR). SAOR measurements operate within the linear viscoelastic (LVE) regime, meaning the material responds proportionally to the applied stress. This makes SAOR analysis well‐supported by established theoretical models. Oscillatory rheometry measurements provide in‐depth mechanical spectroscopy through $G^{\prime }$ and $G^{"}$ measurements, but valid and comparable data across types and datasets of yogurts require standardized geometries and conditions. The ratio of $G^{"}$ to $G^{\prime }$, known as the loss tangent (*δ*), provides insights into the viscoelastic nature of sample. A dominant viscous character is reflected by a high *δ* value ($G^{"}>G^{\prime }$).

**FIGURE 4:**
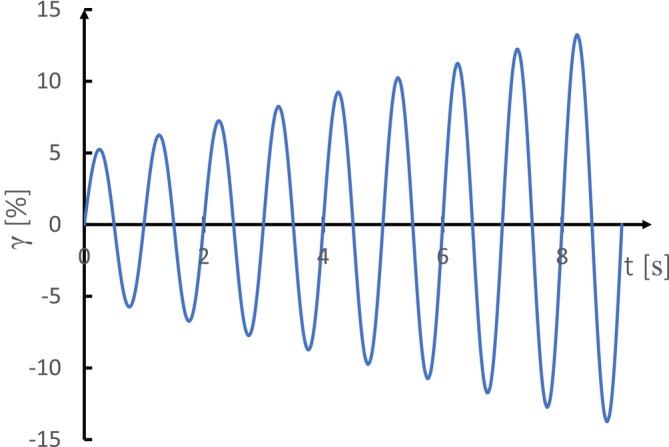
Amplitude sweep with controlled strain.

**FIGURE 5:**
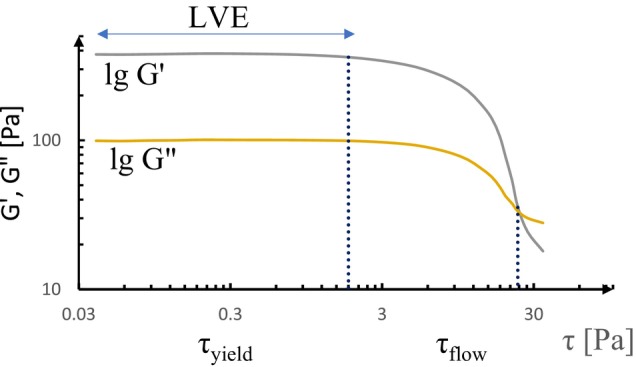
$G^{’}$ and $G^{”}$ moduli in yogurts as functions of stress.

Moreover, SAOR can effectively differentiate between “strong” and “weak” yogurt gels based on the resulting structure. The parameters measured within the LVE region provide valuable insights into the viscoelasticity and overall structure of yogurt gels (Upadhyay and Chen 2020). In an amplitude sweep test, the yield point is identified as the LVE range limit, which is important for understanding the elastic behavior of material. The length of the linear stage indicates the strength of the elastic properties. Figure 4 illustrates the amplitude sweep with controlled strain. This method is effective in capturing important transition points in the structure of yogurt under stress. Additionally, the stress point where it starts to deform is the $\tau_{\text{yield}}$, while flow stress ($\tau_{\text{flow}}$) is the value of shear stress where $G^{\prime }=G^{"}$. Figure 5 shows the determination of $\tau_{\text{yield}}$ and $\tau_{\text{flow}}$.

In contrast, LAOR measurements are performed outside the linear region, probing the behavior of the material at higher strain magnitudes (Upadhyay and Chen 2020). Unlike traditional techniques, LAOR offers independent control over both the applied strain (amplitude) and frequency. This allows researchers to tailor the test to mimic various real‐world scenarios, including those encountered during food processing (Hyun et al. 2011). $\tau_{\text{yield}}$ and yield strain ($\gamma_{\text{yield}}$) are important rheological metrics derived from shear stress–strain curves. $\tau_{\text{yield}}$ marks the beginning of the flow, where shear stress begins to decline (Lucey et al. 1997). Lower $\tau_{\text{yield}}$ indicates a weaker gel network, while decreased $\gamma_{\text{yield}}$ indicates a more brittle or fragile gel structure (Lucey 2001).

A study by Erturk, Bonilla, and Kokini (2021) exemplifies the utility of LAOR in yogurt research. They investigated the LAOR rheological properties of yogurts with different fat content, providing valuable insights into the yogurt structure. The study found a strong correlation between the deformation characteristics of yogurt and its structure under LAOR testing. These findings hold promise for developing more precise yogurt recipes and production processes. By understanding how different ingredients and fat levels affect yogurt texture through LAOR analysis, manufacturers can tailor their products to meet consumer preferences.

**FIGURE 6:**
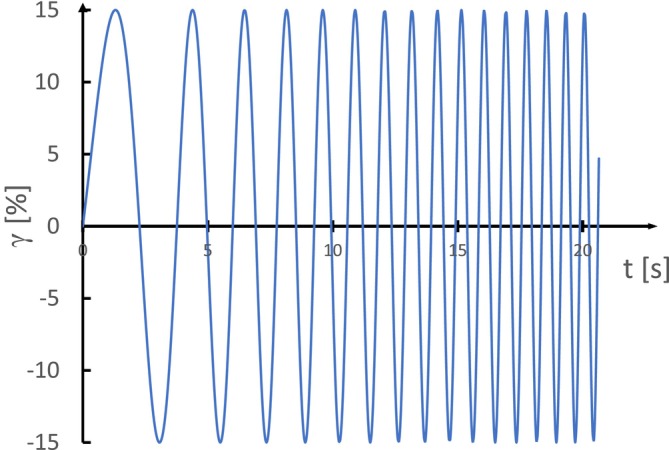
Frequency sweep with controlled strain.

For a better understanding of the elastic behavior of yogurt, a frequency sweep test can be performed, as shown in Figure 6. In this test, a small shear strain value is kept constant while the angular frequency is varied, providing insights into yogurt response to different frequencies of applied stress. Yogurt qualifies as a physical gel due to the increase in dynamic moduli with frequency, and the condition that $G^{\prime }$ consistently exceeds $G^{"}$ during testing (Tunick 2010; Whaley, Templeton, and Anvari 2019). This behavior reflects a balance between repulsive forces like electrostatic repulsions, different cross‐links within the protein network, and attractive forces, such as hydrophobic interactions. This interplay of forces ultimately defines the yogurt gel structure (Lee and Lucey 2010).

**FIGURE 7:**
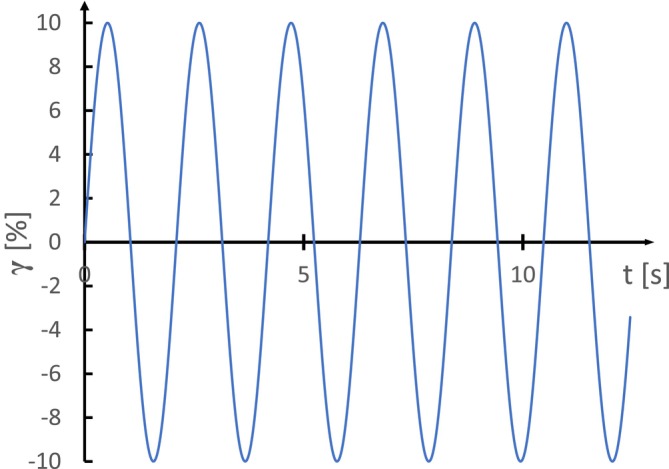
Time sweep with controlled strain.

Moreover, temperature sweep tests evaluate the behavior of material across a range of temperatures, and time sweep tests examine changes in rheological properties over time, as presented in Figure 7. Each of these tests provides valuable information about the viscoelastic properties of material, including its consistency, $G^{\prime }$, and $G^{\prime \prime }$ (Eroglu et al. 2015; Ramaswamy, Chen, and Rattan 2015).

The back extrusion test is an empirical method used to characterize the flow behavior of yogurt by applying substantial forces through a simple geometry and procedure. In this method, a cylindrical probe is inserted into the sample and then withdrawn at a constant velocity while the force exerted on the probe is recorded. One key advantage of this technique is its ability to evaluate food products within their original containers, thereby preserving the microstructure. However, consistency in geometry, testing parameters, and sample quantity is crucial for accurate results. When samples are transferred to different containers, care must be taken to avoid damaging the microstructure or causing excessive compaction. Additionally, highly adhesive samples can introduce measurement artifacts, such as a vacuum forming beneath the probe during extraction, which can affect the results. For instance, Yu, Wang, and McCarthy (2016) used back extrusion tests to compare the texture of various yogurt types, including fat‐free, fat‐free with milk solids nonfat (MSNF), whole fat, and whole fat with MSNF yogurts. Their findings indicated that adding MSNF significantly increased the peak values in the compression phase, showing that protein content has a more substantial impact on yogurt texture than fat content. Yogurts with MSNF exhibited higher firmness, adhesive force, and adhesiveness, with whole fat with MSNF yogurt showing the highest values. The adhesive properties of yogurts with MSNF were significantly different from those without it.

**FIGURE 8:**
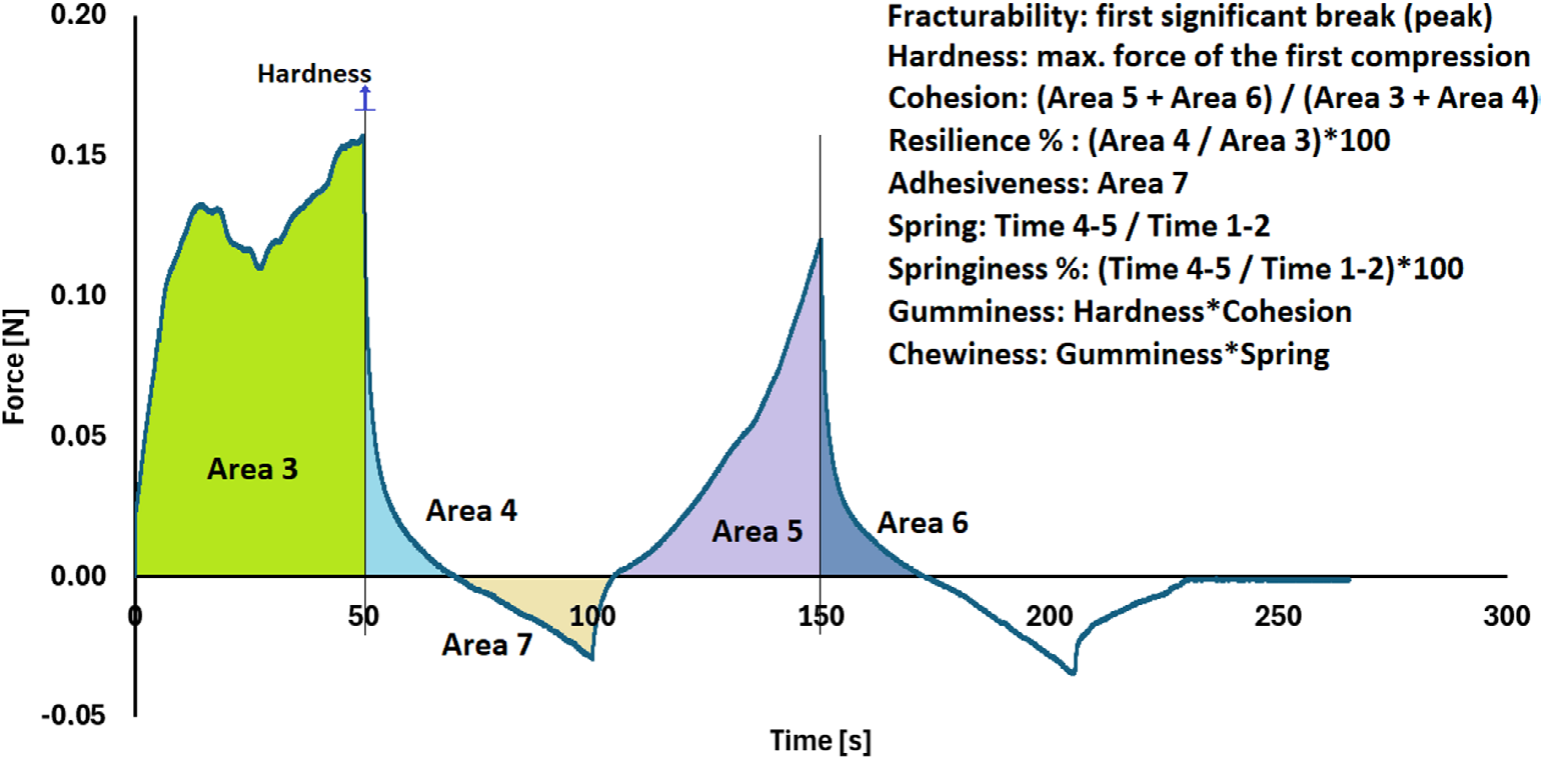
Texture profile analysis of yogurt. *Source:* Self‐measured.

Texture profile analysis is another widely used technique using a texturometer that applies dual compression to a sample to quantify rheological properties such as fracturability, firmness, cohesiveness, adhesiveness, springiness, gumminess, and chewiness. TPA involves recording the force‐time profile during the compression‐relaxation cycle, where the instrument crosshead descends vertically, deforming a cylindrical sample placed between a flat plate and a lower platform. The crosshead then retracts at the same rate, simulating a biting action. This method provides valuable insights into overall texture and allows for comparisons among multiple samples. Figure 8 presents the TPA diagram of yogurt. For example, Mudgil, Barak, and Khatkar (2017) explored how partially hydrolyzed guar gum (PHGG), culture levels, and incubation time affect yogurt texture using TPA combined with response surface methodology. They found that adding PHGG decreased firmness and gumminess but increased adhesiveness, cohesiveness, and springiness. The culture level had little effect, while longer incubation times negatively impacted the texture. The optimal yogurt texture was achieved with 3.37% PHGG, 1.96% culture, and 5.96 h of incubation.

## 7 Yogurt Microstructure

Textural and rheological measurements provide valuable insights into the microscopic structure and interactions within a yogurt gel network (Rao 2007). The rheological properties of this gel are directly linked to the interactions between protein building blocks, the connections between these strands, and the overall abundance of strands that contribute to a cohesive network (Lucey 2016). The physical transformation of milk into a semi‐solid or solid state is known as milk gelation, characterized by a shift from a low‐viscosity Newtonian fluid (Foegeding and Davis 2011). When milk is acidified, the arrangement of milk proteins undergoes significant changes, leading to the formation of a porous protein network that traps the whey (Lucey 2004). The effect of denatured whey protein from milk on protein network density and gel characteristics in set and stirred gels depends on the applied heat treatment.

The microstructure of yogurts can be influenced by processing parameters. Erturk, Bonilla, and Kokini (2021) conducted a study on the effect of fat reduction on yogurt microstructure using both SEM and cryo‐SEM techniques. They analyzed two commercial yogurt brands with different fat contents. SEM images showed that higher fat content in the yogurt sample led to a decrease in the size of casein micelle aggregates while improving their overall contact area, resulting in a uniform and well‐connected network. Replacing fat with whey protein microparticles led to the formation of larger protein aggregates. In addition, Cryo‐SEM analysis revealed that the first brand exhibited a dense, cohesive protein network with tightly packed casein and whey protein micelles, creating a well‐connected structure. In contrast, the second brand showed a looser, honeycomb‐like structure with larger voids and finer polysaccharide‐lined walls, creating a more irregular network with higher lacunarity.

Torres et al. (2018) applied confocal laser scanning microscopy to investigate the microstructural characteristics of stirred low‐fat yogurt (with 0.5% fat content) containing MWPs. They found that replacing fat with whey protein microparticles led to the formation of larger protein clusters. This resulted in a less compact, more dispersed network with wider gaps between protein clusters in low‐fat yogurts. Similarly, Sandoval‐Castilla et al. (2004) investigated the effects of three different fat replacers—WPC, MWP, and modified tapioca starch—on the microstructure and texture of yogurt. They used SEM to assess the microstructure of the yogurt samples. Their findings revealed that the type of fat replacer had a significant effect on the microstructure and textural properties of yogurt.

WPC yogurt formed chain‐like structures of casein micelles, while MWP yogurt maintained a spatial distribution similar to reduced‐fat yogurt, with MWP embedded within the network. Modified tapioca starch yogurt exhibited the most disorganized structure, with solubilized starch molecules dispersed throughout the casein micelle network and starch gel fragments forming distinct entities. WPC‐based yogurts and blends of WPC and MWP produced yogurts with textural profiles closely resembling full‐fat yogurts, while MWP yogurts displayed lower tension, firmness, and adhesiveness but higher cohesiveness.

**FIGURE 9:**
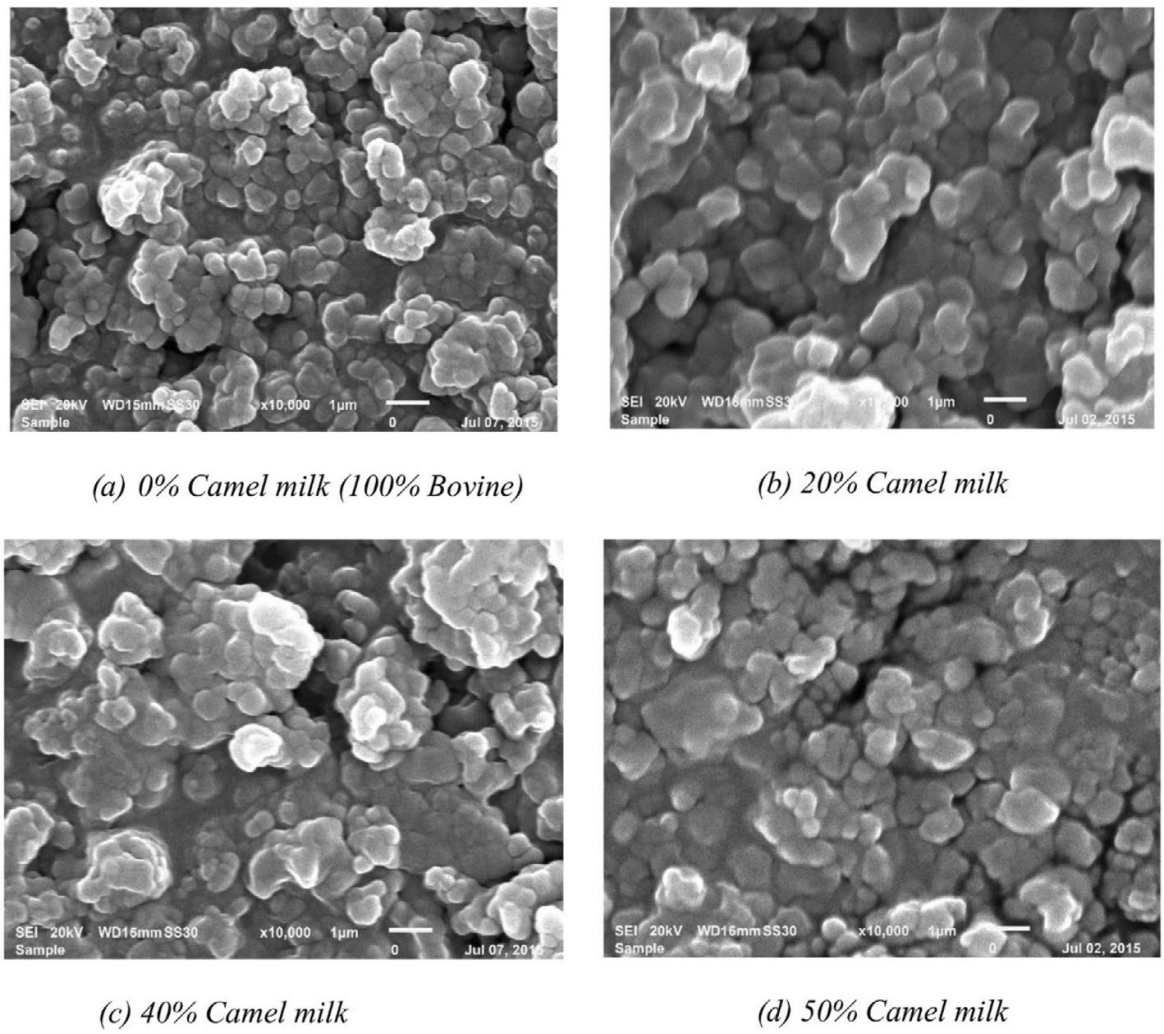
SEM images of camel and bovine milk yogurts at 10,000× magnification: (a) 0%, (b) 20%, (c) 40%, and (d) 50% camel milk, reprinted from (Kamal‐Eldin et al. 2020) with permission from Elsevier.

Kamal‐Eldin et al. (2020) studied the rheology and microstructural characteristics of yogurts made from bovine and camel milk. As shown in Figure 9, SEM images reveal that incorporating camel milk into bovine milk results in larger, less well‐defined casein micelles. Pure cow milk yogurt produced distinct, tiny particles, while camel milk blends yielded larger, softer clusters with less obvious interparticle spaces.

Lee and Lucey (2003) used confocal scanning laser microscopy to investigate the structure of acid milk gels. The presence of larger pores in weaker regions indicates that the casein particle connections are fragile, potentially reducing the fracture stress of the acid milk gel. Marafon et al. (2011), using SEM, found that fortified yogurt exhibited a more compact matrix compared to non‐fortified yogurt, likely due to higher protein content. Processing conditions, such as heating and smoothing temperatures, significantly impact the microstructure and textural properties of yogurt. Gregersen et al. (2021) discovered that additional heating of the milk matrix resulted in a more uniform and branched network structure, accounting for the observed increase in $G^{\prime }$. Gilbert et al. (2020) used laser diffraction and optical microscopy to study the size and distribution of microgels in stirred yogurt over time, finding that the smoothing temperature had a more significant effect on microgel size than the type of whey protein ingredient.

Modler and Kalab (1983) used SEM and transmission electron microscopy to investigate the microstructure of yogurt stabilized with various whey protein‐ and casein‐based ingredients. They found that yogurt microstructure is highly dependent on the types of milk protein used. Casein micelles in yogurts with casein were extensively fused, forming long chains with short inter‐micelle bonds, while yogurts with WPC and skim milk powder exhibited individual casein micelles connected by weaker links. SC induced the production of large, heavily fused micelles. Yogurts with different commercial WPCs shared a similar structure characterized by individual casein micelles interspersed with flocculated protein, differing significantly from casein‐based yogurts.

The η of yogurt is influenced by the volume fraction of microgels, determined by microgel shape and size, and the viscosity of the surrounding liquid (serum) (Loewen, Nöbel, and Hinrichs 2017). Increased microgel volume fraction and serum viscosity contribute to higher viscosity. As shear intensity increases, yogurt microgels decrease in size and increase in sphericity, leading to a reduction in overall viscosity (Walstra et al. 2005). This relationship between shear intensity and microgel size was confirmed by van Marle et al. (1999) and Javanmard et al. (2018). van Marle et al. (1999) developed a micro‐rheological model linking yogurt viscosity to its microstructure, particularly microgel properties such as porosity, network rigidity, elasticity, and the dynamic interactions between protein aggregates.

In summary, the application of advanced microscopy techniques has revolutionized our understanding of yogurt microstructure and its relationship with rheological properties. Leveraging these insights, dairy product manufacturers can develop improved yogurt formulations with enhanced texture, stability, and sensory appeal, meeting the evolving demands of consumers for high‐quality dairy products.

## 8 Conclusion and Future Outlook

Understanding yogurt rheology is essential for optimizing its texture, stability, and sensory properties. This knowledge enables manufacturers to create products that align with consumer preferences, including plant‐based, low‐fat, high‐protein, or drinking varieties, while maintaining consistency and shelf‐life stability through enhanced quality control.

Despite extensive research on the rheological characteristics of yogurt, further research should focus on developing non‐destructive methods to monitor rheological changes during production and packaging, as well as exploring the effects of extended storage on yogurt rheology, as most studies focus on a limited expiration date of up to 30 d. Additionally, advanced microscopy and spectroscopy techniques offer valuable insights into the microscopic and nanoscopic structures of yogurt, supporting the development of detailed models to predict and enhance texture stability over storage. Finally, computational modeling offers exciting potential to simulate and predict the impact of various processing factors on yogurt rheology, accelerating product innovation and quality assurance.

**Nomenclature**
η
: Apparent viscosity (Pas)
$\eta^{*}$
: Complex viscosity (Pas)
$G^{\prime }$
: Storage modulus (Pa)
$G^{"}$
: Loss modulus (Pa)
TS: Total solid (%)
SMP: Skimmed milk powder
SM: Skimmed milk
EPS: Exopolysaccharide
WPI: Whey protein isolate
WPC: Whey protein concentrate
MWP: Microparticulated whey protein
UV‐C: Ultraviolet C
HPP: High‐pressure processing
TPA: Texture profile analysis
SAOR: Small amplitude oscillatory rheology
LAOR: Large amplitude oscillatory rheology
LVE: Linear viscoelastic

## Author Contributions

**Thong Le Ba:** conceptualization, writing – original draft, visualization. **Mai Sao Dam:** formal analysis, writing – review and editing, validation. **Lien Le Phuong Nguyen:** validation, formal analysis, writing – original draft. **Tímea Kaszab:** conceptualization, writing – original draft, writing – review and editing, supervision. **László Baranyai:** supervision, writing – review and editing.

## Ethics Statement

This study does not involve any human or animal testing.

## Conflicts of Interest

The authors declare no conflicts of interest.

## Data Availability

Data sharing not applicable to this article as no datasets were generated or analysed during the current study.
